# Estrogen-sensitive activation of SGK1 induces M2 macrophages with anti-inflammatory properties and a Th2 response at the maternal–fetal interface

**DOI:** 10.1186/s12958-023-01102-9

**Published:** 2023-05-24

**Authors:** Yiyun Lou, Zhujing Fu, Ye Tian, Minhao Hu, Qijing Wang, Yuanyuan Zhou, Ning Wang, Qin Zhang, Fan Jin

**Affiliations:** 1 Department of Gynaecology, Hangzhou TCM Hospital Affiliated to Zhejiang Chinese Medical University, Hangzhou, 310007 China; 2 Medical Department, Jinhua Municipal Central Hospital, Jinhua, 321000 China; 3 Medical School, Zhejiang Chinese Medical University, Hangzhou, 310053 China; 4 Yangtze River Delta Center for Drug Evaluation and Inspection of National Medical Products Administration, Shanghai, 201210 China; 5 Department of Reproductive Endocrinology, Women’s Hospital, School of Medicine, Zhejiang University, Hangzhou, 310006 China; 6 Key Laboratory of Reproductive Genetics, Women’s Reproductive Healthy Laboratory of Zhejiang Province, Women’s Hospital, Zhejiang University, Hangzhou, 310006 China

**Keywords:** Abortion, Spontaneous, Serum-glucocorticoid regulated kinase, Estradiol, Macrophages, Decidua, T-Lymphocytes, Helper-inducer

## Abstract

**Background:**

Decidual macrophages participate in immune regulation at the maternal–fetal interface. Abnormal M1/M2 polarization of decidual macrophages might predispose immune maladaptation in recurrent pregnancy loss (RPL). However, the mechanism of decidual macrophage polarization is unclear. We explored the role of Estradiol (E_2_)-sensitive serum-glucocorticoid regulated kinase (SGK) 1 in promoting macrophage polarization and suppressing inflammation at the maternal–fetal interface.

**Methods:**

We assessed serum levels of E_2_ and progesterone during first trimester of pregnancy in women with or without threatened miscarriages (ended in live birth, *n* = 448; or early miscarriages, *n* = 68). For detection of SGK1 in decidual macrophages, we performed immunofluorescence labeling and western blot analysis applying decidual samples from RPL (*n* = 93) and early normal pregnancy (*n* = 66). Human monocytic THP-1 cells were differentiated into macrophages and treated with Toll-like receptor (TLR) 4 ligand lipopolysaccharide (LPS), E_2_, inhibitors or siRNA for in vitro analysis. Flow cytometry analysis were conducted to detect macrophages polarization. We also applied ovariectomized (OVX) mice with hormones exploring the mechanisms underlying the regulation of SGK1 activation by E_2_ in the decidual macrophages in vivo.

**Results:**

SGK1 expression down regulation in the decidual macrophages of RPL was consistent with the lower concentration and slower increment of serum E_2_ from 4 to 12 weeks of gestation seen in these compromised pregnancies. LPS reduced SGK1 activities, but induced the pro-inflammatory M1 phenotype of THP-1 monocyte-derived macrophages and T helper (Th) 1 cytokines that favored pregnancy loss. E_2_ pretreatment promoted SGK1 activation in the decidual macrophages of OVX mice in vivo. E_2_ pretreatment amplified SGK1 activation in TLR4-stimulated THP-1 macrophages in vitro through the estrogen receptor beta (ERβ) and PI3K pathway. E_2_-sensitive activation of SGK1 increased M2 macrophages and Th2 immune responses, which were beneficial to successful pregnancy, by inducing *ARG1* and *IRF4* transcription, which are implicated in normal pregnancy. The experiments on OVX mice have shown that pharmacological inhibition of E_2_ promoted nuclear translocation of NF-κB in the decidual macrophages. Further more, pharmacological inhibition or knockdown of SGK1 in TLR4-stimulated THP-1 macrophages activated NF-κB by promoting its nuclear translocation, leading to increased secretion of pro-inflammatory cytokines involved in pregnancy loss.

**Conclusion:**

Our findings highlighted the immunomodulatory roles of E_2_-activated SGK1 in Th2 immune responses by priming anti-inflammatory M2 macrophages at the maternal–fetal interface, resulting in a balanced immune microenvironment during pregnancy. Our results suggest new perspectives on future preventative strategies for RPL.

**Supplementary Information:**

The online version contains supplementary material available at 10.1186/s12958-023-01102-9.

## Background

Recurrent pregnancy loss (RPL) is defined as two or more spontaneous losses of a pregnancy before 20–24 weeks of gestation [1], which is experienced by approximately 2.5% of women trying to conceive [2]. A multifactorial origin of this reproductive phenotype has been proposed [3], among which maternal immunological aberration is postulated to be one of the main culprits [4]. The immune system is one of the major players in maternal–fetal crosstalk [5]. Indeed, the decidua is an immunologically privileged spot at the maternal–fetal interface that links the maternal and fetal immune responses, as well as recognizing the semi-allogenic fetus, thereby maintaining the pregnancy [6]. The immune cells residing in the decidua play important roles in the maintenance of pregnancy [7]. Among these immune cells, macrophages comprise the second most predominant leukocyte population in the decidua, with relatively constant numbers throughout gestation [8]. Macrophages can be polarized into M1 and M2 phenotypes [9]. M1 macrophages are potent effector cells that eliminate external pathogens and induce pro-inflammatory T helper (Th) 1 cytokines, such as interleukin (IL)-12, tumor necrosis factor-$\alpha$ (TNF-$\alpha$), and IL-6 [10]. By contrast, M2 macrophages attenuate these Th1 responses by producing anti-inflammatory Th2 cytokines, such as IL-4, IL-5, and IL-10 [10]. The macrophage polarization induced Th1/Th2 immune response plays an important role in the occurrence of spontaneous abortion [11]. An excessive pro-inflammatory Th1 immune response is associated with pregnancy failure, while a Th2 immune response downregulates inflammatory and cytotoxic pathways by selectively promoting angiogenesis to protect embryos from maternal immune rejection and maintain normal pregnancy [11]. Studies have shown that M2-type polarization of decidual macrophages enriched at the maternal–fetal interface would be conducive to maintaining a balanced immune microenvironment during pregnancy, thus promoting the immune tolerance of semi-allograft embryos, remodeling local tissues and blood vessels, and other affecting pregnancy-related physiological processes [12]. By contrast, M1-type polarization of macrophages in the decidua can trigger local inflammatory immune responses, leading to premature birth and other adverse pregnancy outcomes [13]. Thus, the ideal intrauterine microenvironment during early pregnancy is generally thought to be mainly an immuno-suppressive M2–Th2 immune response.

Toll-like receptors (TLRs), primarily TLR4, play an important role in Th1/Th2 immune responses [14]. TLR4 is one of the best characterized innate immune receptors and can induce excessive macrophage inflammatory responses [15]. TLR4 activation, for example by lipopolysaccharide (LPS), plays a pivotal role in early gestation [16]. LPS was used to activate TLR4 in vivo and in vitro, contributing to the accelerated senescence of the placenta [17]. TLR4-mediated cytokine production increased aberrant pro-inflammatory responses at the maternal–fetal interface, creating a hostile environment for the developing embryo, and contributing to early miscarriage and preeclampsia [18, 19]. Inflammatory mechanisms are normally tightly regulated throughout pregnancy; however, triggering impaired local immune/inflammatory responses in gestational tissues could negatively affect pregnancy and result in pregnancy complications [20]. In a study of the prevention of RPL, Zou et al*.* found that blocking abnormal TLR4 activation, and its downstream nuclear factor-kappa B (NF-κB) signaling pathway at the maternal–fetal interface, led to an improved pregnancy [21]. Moreover, in studies on the critical roles of trophoblasts in the pathogenesis of RPL, LPS-induced M1 macrophage polarization suppressed trophoblast migration and invasion [13, 22]. Interestingly, 17ß-estradiol (E_2_) has been reported to attenuate LPS-induced elevation of TLR4 and macrophage inflammation [23, 24]. Recently, E_2_ was demonstrated to facilitate the resolution of pro-inflammation conditions by enhancing the polarization of M1 to M2 macrophages, thereby conferring cardioprotection [25]. However, the impact of E_2_ on immune cells is controversial. A study has shown that E_2_ promotes the pro-coagulatory and pro-inflammatory responses of monocytes elicited by LPS in the case of RPL associated with extended persistence of antiphospholipid antibodies [26]. E_2_ plays an intricate and essential role during pregnancy by helping to maintain the immune balance through regulatory T cells (Tregs) regulation to improve pregnancy outcome in infection-induced abnormal pregnancy [27]. In a mouse model, Park et al. identified E_2_ regulated genes that are critical for successful embryo implantation by controlling cell signaling involved in epithelial cell remodeling [28]. E_2_ has been suggested to prevent allogeneic fetal rejection by upregulating transforming growth factor beta (TGF‑ß) expression in decidual tissues and chorionic villi during pregnancy, and might be used as an immunomodulatory [29]. Therefore, the effects of E_2_ on TLR4 signaling in decidual macrophages for the immunomodulation of the maternal–fetal interface are unclear and require further investigation.

Serum-glucocorticoid regulated kinase (SGK) 1 can be directly activated by E_2_ via the estrogen receptor (ER), and acts as an LPS/TLR4 signal transduction target [30, 31]. SGK1 has been implicated in macrophage polarization and inflammatory processes [32]. Emerging evidence points to a role of SGK1 in immunomodulation [33]. Importantly, SGK1 downregulation has been implicated in RPL, in which it might sensitize endometrial stromal cells to oxidative cell death, thereby predisposing women to early spontaneous miscarriages [34]. Moreover, enhanced phosphoinositide 3-kinase (PI3K)-SGK1 activities might be involved in embryo implantation [35]. Our previous study found that downregulation of SGK1 expression in decidual tissue affected endometrial decidualization, resulting in embryo implantation failure [36]. We found that decidual SGK1 affects the apoptosis of decidual stromal cells (DSCs) in vitro, and increase the amount of Th2 cytokines secreted by DSCs [37]. However, the role of SGK1 in intrauterine immune maladaptation associated with RPL remains to be determined. There are still significant gaps in our understanding of whether and how SGK1 transduces the E_2_ stimulus to modulate TLR-induced innate immune responses of macrophages at the maternal–fetal interface during early pregnancy.

In the present study, we examined the concentrations of serum E_2_ during the early pregnancy from the 4^th^–12^th^ week of gestation, and analyzed the activation of decidual SGK1 in women suffering from RPL. Then, we employed THP-1 human monocyte-derived macrophages and ovariectomized mice to explore the correlation between SGK1 activation in decidual macrophages and the maintenance of normal healthy pregnancy.

## Materials and methods

### Human sample collection

The study was approved by both the Medical Ethics Committee of Hangzhou Hospital of Traditional Chinese Medicine, Zhejiang, China (approval numbers: 2014LL077, 2018KY022, 2018KY056) and Institutional Review Board of the School of Medicine, Zhejiang University, China (approval numbers: 20120019, 20130044). All participants in this study (from March 2012 to November 2021) provided written informed consent. All human experiments were conducted in accordance with approved regulations and the principles of Declaration of Helsinki. Blood samples were obtained from healthy pregnant women and pregnant women threatened with miscarriages (ended in live birth, *n* = 448; or early miscarriages, *n* = 68). Human decidual tissue samples were obtained from physically normal pregnant women that were legally terminated for non-medical reasons (*n* = 66) and because of RPL (excluding genetic abnormalities, endocrine disruptions, anatomical defects, infective diseases, and patients unwilling to sign informed consent, *n* = 93) in the first trimester of gestation. The intrauterine pregnancies and gestational weeks were confirmed using both ultrasound scans and menstruation cycle determination. Supplementary Table S1 and S2 describe the comparative demographic characteristics of the study population. Fresh decidual tissues were transported to our laboratory within 1 h after termination. Following constant stirring in phosphate-buffered saline (PBS) to remove most of the remaining blood, the decidual tissues were stored in ice-cold Dulbecco’s modified Eagle’s medium (DMEM)/F12 (Gibco, Grand Island, NY, USA) for subsequent processing. Then, a portion of the decidual tissues were fixed in 4% paraformaldehyde (E672002-0100, Sangon Biotech, Shanghai, China)-PBS and embedded in paraffin wax. The paraffin-embedded tissue blocks were then sectioned (4 μm thick).

### Animals and interventions

The animal experiments were approval by the Animal Ethics Committee of Zhejiang Chinese Medical University (approval number: 20211018–07, 20211129-19), and were conducted at Zhejiang Chinese Medical University Laboratory Animal Research Center in accordance with the Guide for the Care and Use of Laboratory Animals (China). Female Institute of Cancer Research (ICR) mice (8–10 weeks old, 20 ± 5 g) were purchased from the SIPPR/BK laboratory animal company (Shanghai, China). The mice were raised under a 12 h light/dark cycle under specific pathogen-free conditions with 55–65% relative humidity at 22–24°C, and were allowed free access to water and standard rodent chow. Mice were ovariectomized (OVX) under general anesthesia, as described previously [38], and were kept individually for two weeks for complete recovery. The mice were then regrouped again, and 60 mice were randomly divided into six groups with 10 mice in each group: (1) The E_2_ group: OVX mice received daily intraperitoneal (IP) injections of corn oil containing E_2_ (100 ng of E_2_ in 100 μL of corn oil/mouse, E2758, Sigma-Aldrich, St. Louis, MO, USA) at 8:30 a.m. for 3 days [39]. (2) The progesterone (P_4_) group: OVX mice were injected with corn oil containing P_4_ (1 mg of P_4_ in 100 μL of corn oil/mouse, Cat. No. 2835, Tocris Bioscience, Bristol, UK) at 8:30 a.m. for 3 consecutive days [40]. (3) The E_2_ + P_4_ group: OVX mice received daily IP injection of E_2_ (100 ng of E_2_ in 100 μL of corn oil/mouse) at 8:30 a.m. for 2 days, and then were treated with P_4_ (1 mg of P_4_ in 100 μL of corn oil/mouse) by IP injection at 8:30 a.m. on the 3rd day [38]. (4) The ICI 182780 group: female mice with intact ovaries received IP injection of ICI 182780 (an estrogen receptor antagonist, 100 μg/mouse, Cat. No. 1047, Tocris Bioscience) at 08:30 a.m. on the first day [41]. (5) OVX control group: OVX mice were injected with corn oil (100 μL corn oil/mouse, 8001–30-7, Picasso-e, Shanghai, China) alone for 3 consecutive days. (6) Sham group: female mice with intact ovaries; all mice were euthanized by cervical dislocation at 6:00 p.m. on Day 3. Uteruses were collected and stored in PBS.

### Hormone analyses

The determinations of the hormones E_2_ and P_4_ were performed using an electrochemiluminescence immunoassay (Cobas e601, Roche Diagnostics, Basel, Germany) with Roche’s Cobas reagents and a competitive chemiluminescent enzyme immunoassay (Immulite 2000, Siemens Medical Solutions Diagnostic, München, Germany). The intra-assay coefficients of variation for E_2_ and P_4_ were 5.007.14%, and 3.754.84%, respectively. The inter-assay coefficients variation for E_2_ and P_4_ were 1.244.84%, and 0.415.20%, respectively.

### Immunofluorescence labeling and confocal microscopy

For labeling, decidual tissue sections were incubated with primary antibodies: mouse monoclonal anti-SGK1 antibodies (1:50, SC-28338, Santa Cruz Biotechnology, Santa Cruz, CA, USA), rabbit monoclonal anti-CD68 antibodies (1:500, 76437, Cell Signaling Technology, Danvers, MA, USA) overnight at 4°C. The slides of THP-1 cells before and after phorbol 12-myristate 13-acetate (PMA; Sigma-Aldrich, P1585) pretreatment were incubated with anti-CD68 antibodies at 4°C overnight. Then, the sections of human decidual tissue and THP-1 macrophages were incubated with Alexa Fluor® 488 Donkey Anti-Rabbit IgG (1:400, ab150073, Abcam, Cambridge, UK) and Alexa Fluor® 647 Donkey Anti-Mouse IgG (1:400, ab150107, Abcam, UK) secondary antibodies at 37°C for 1 h in the dark. The nuclei were stained with 4’,6-diamidino-2-phenylindole (DAPI, C1005, Beyotime Biotechnology, Shanghai, China) and washed using PBS (3 × 5 min). The slices were photographed and results were recorded using a laser confocal microscope (LSM 710, Zeiss, Wetzlar, Germany).

### Isolation of mice decidual macrophages

Decidual macrophages were isolated following a previously described protocol with modifications [42, 43]. Decidual tissues were washed with sterile PBS and crushed into small pieces. After digestion in 3 mg/ml collagenase Type IV (C4-28, Sigma-Aldrich), and 100 IU/ml DNase I (10104159001, Sigma-Aldrich) for 30 min at 37°C, the cell suspensions were filtered through a 70 μM nylon mesh strainer. Decidual macrophages were obtained by using an EasySep™ Mouse F4/80 Positive Selection Kit (100–0659, STEMCELL Technologies, Vancouver, Canada) according to the manufacturer’s instructions. After cell acclimatation, decidual macrophages were fixed in 4% paraformaldehyde-PBS. Then, the decidual macrophages of OVX mice were incubated with rabbit polyclonal anti-CD14 antibodies (1:100, 17000-1-AP, Proteintech) at 4°C overnight, and then with Alexa Fluor® 488 Donkey Anti-Rabbit IgG (1: 400, ab150073, Abcam). The purity of the enriched decidual macrophages reached approximately 90%.

### Cell culture and in vitro treatments

Human monocytic THP-1 cells (SCSP-567) were purchased from the Type Culture Collection of the Chinese Academy of Sciences, Shanghai, China, and maintained in Roswell Park Memorial Institute Medium 1640 (RPMI-1640, SH30809.01B, Hyclone, Logan, UT, USA) supplemented with 1% streptomycin-penicillin (CORNING Inc., Corning, NY, USA, 30–002-CIa) and 10% fetal calf serum (FCS, 16000-044, Gibco, Grand Island, NY, USA). THP-1 cells (1 × 10^6^) were seeded with 120 ng/ml PMA overnight. Afterwards, THP-1 monocyte-derived macrophages were confirmed by immunofluorescence analysis according to CD68 (sc-9139, Santa Cruz) positivity. Macrophages were plated in 24-well microplates and incubated in phenol red-free RPMI-1640 (90022-500, Solarbio, Beijing, China) containing 10% charcoal-filtered Fetal Bovine Serum (FBS, 04–201-1A, Biological Industries, Kibbutz Beit-Haemek, Israel) and 1% streptomycin-penicillin in an atmosphere containing 5% CO_2_ and 100% humidity at 37°C for 24 h. Then, the macrophages were supplemented with stimulators combined with or without inhibitors: (1) The control group only received medium, (2) the LPS (TLR4 ligand) group were treated with 1 μg/ml LPS (L4391, Sigma-Aldrich, Merck Kgaa, Darmstadt, Germany), (3) the E_2_ group received 1 μg/ml LPS + 10 nM E_2_ (E2758, Sigma-Aldrich), (4) the LY294002 (PI3K signaling pathway inhibitor) group received 1 μg/ml LPS + 10 nM E_2_ + LY294002 (25 μM, 9901S, Cell Signaling Technology), (5) the GSK650394 (SGK1 inhibitor) group received 1 μg/ml LPS + 10 nM E_2_ + GSK650394 (10 μM, S7209, Selleck, Houston, TX, USA), (6) the BAY 11–7082 (NF-κB inhibitor) group received 1 μg/ml LPS + 10 nM E_2_ + 10 μM GSK650394 + BAY 11–7082 (30 μM, S2913, Selleck), (7) the ICI 182780 (ER antagonist) group received 1 μg/ml LPS + 10 nM E_2_ + ICI 182,780 (1 μM, Cat. No. 1047, Tocris Bioscience), (8) the 1,3-bis(4-hydroxyphenyl)-4-methyl-5-[4-(2-piperidinylethoxy)-phenol]-1H-pyrazole dihydrochloride (MPP, ERα antagonist) group, 1 μg/ml LPS + 10 nM E_2_ + MPP (1 μM, Cat. No. 1991, Tocris Bioscience), and (9) the 4-[2-Phenyl-5,7-bis(trifluoromethyl)pyrazolo[1,5-a]pyrimidin-3-yl]phenol (PHTPP; Cat. No. 2662, Tocris Bioscience, an ERβ antagonist) group received 1 μg/ml LPS + 10 nM E_2_ + 1 μM PHTPP for another 24 h. The supernatants and macrophages were collected for subsequent assays.

### Quantitative real time reverse transcription RT-RCR (qRT-PCR) analysis

Total RNA was extracted from decidual tissues and cell culture using Trizol (Invitrogen, Waltham, MA, USA), and reverse transcription was carried out using a YBR PrimeScript RT-PCR Kit (Takara, Dalian, China) according to the manufacturer’s protocol. The quantitative real-time PCR (qPCR) step of the qRT-PCR protocol was carried out using the cDNA as the template and the SYBR Premix Ex Taq (Takara) on an ABI 7900 thermocycler (Foster City, CA, USA) as recommended by the manufacturer. Relative mRNA expression values were normalized to human GAPDH (encoding glyceraldehyde-3-phophate dehydrogenase) expression. The results were analyzed using the △cycle threshold (CT) method [44]. The primers used for qPCR amplification of the cDNA are shown in Supplementary Table S3. All primers were synthesized by Invitrogen Corporation (Shanghai, China).

### Western blotting analysis

Total proteins from decidual tissues, cultured THP-1 cells, or cells of mouse decidual macrophages, were extracted using Radioimmunoprecipitation assay (RIPA) lysis Buffer (89900, Thermo Fisher Scientific, Waltham, MA, USA), and quantified using a bicinchoninic acid (BCA) protein assay kit (P0010, Beyotime Biotechnology, Jiangsu, China). About 60 μg of protein was subjected to 8% sodium dodecyl sulfate–polyacrylamide gel electrophoresis (SDS-PAGE), and then transferred to a polyvinylidene fluoride (PVDF) membrane (IPVH00010, Millipore, Billerica, MA, USA). After blocking with 5% skimmed milk, the membranes were incubated with the primary antibodies (Supplementary Table S4) overnight at 4°C. NE-PER™ Nuclear and Cytoplasmic Extraction Reagents (78835, Thermo Fisher Scientific) was used to extract nuclear and cytoplasmic proteins from cell culture. Antibodies recognizing β-actin and anti-TATA binding protein (TBP) antibodies were used as loading controls. After incubation with primary antibodies, the membranes were incubated with secondary antibodies: goat anti-mouse IgG-horseradish peroxidase (HRP) (1:5000, 31160, or 31431, Thermo Fisher Scientific) and goat anti-rabbit IgG-HRP (1:5000, 31210, Thermo Fisher Scientific) for 1 h at room temperature. The immunoreactive protein bands were visualized using SuperSignal West Dura Extended Duration Substrate (34075, Thermo Fisher Scientific). Band intensities were analyzed using Image J 1.8.0 software (NIH, Bethesda, MD, USA). Data are presented as the ratio of the optical density of the target protein to the internal controls on the same blot.

### Immunoassay for cytokines

After stimulation and sample treatments, the cell culture supernatants were collected and analyzed for IL-4, IL-5, interferon gamma (IFN-γ), IL-12p70, TNF-α, and IL-6 secretion using a microsphere-based ProcartaPlex® Multiplex Immunoassay (EPX060-10009–901, eBioscience/Affymetrix, Frankfurt, Germany) and subsequently analyzed on Luminex test equipment (Bio-Plex®System (Bio-Rad, Munich, Bavaria, Germany) according to manufacturer’s instructions.

### Flow cytometry analysis

Antibodies for the flow cytometry assay were purchased from Biolegend (San Diego, CA, USA): allophycocyanin (APC)-conjugated anti-human CD163 (326509), phycoerythrin (PE)-conjugated anti-human CD80 (305207) or anti-human CD206 (321105), Peridinin chlorophyll protein complex (PerCP)/Cy5.5 anti-human CD86 (305419). After stimulation and inhibition for 48 h, THP-1 monocyte-derived macrophages (1 × 10^6^) in 50 μl of PBS were incubated with the fluorescence-labeled antibodies for 30 min at 4 °C in the dark. The expression of cell surface molecules was measured using FACSVerse (BD Biosciences, San Jose, CA, USA) according to manufacturer’s instructions. Mouse IgGs of the same isotype served as negative controls, incubated in equivalent immunostaining conditions.

### RNA interference

The transient knockdown assay was performed using small interfering RNA (siRNA) targeting human *SGK1* (sense: GGAGCUGUCUUGUAUGAGAdTdT; antisense: UCUCAUACAAGACAGCUCCdTdT), *ESR2* (estrogen receptor 1) (sense: GCCCUGCUGUGAUGAAUUAdTdT; antisense: UAAUUCAUCACAGCAGGGCdTdT) and non-targeting negative control siRNA (Biomics, Jiangsu, China). THP-1 monocyte-derived macrophages were cultured to 70–80% confluence in serum-free medium, and then transfected with 40 nM siRNA using RNAiMAX (Life Technologies, Carlsbad, CA, USA) for 48 h. Macrophages were then incubated with E_2_ overnight and harvested for subsequent analysis.

### Data analysis

Statistical analysis was performed using SPSS 16.0 (IBM Corp., Armonk, NY, USA). Student’s *t* tests were employed to assess the difference between two groups. For the analysis of multiple groups, one-way analysis of variance with least squares difference (LSD), Dunnett's test, and Student–Newman–Keuls (SNK) post hoc comparison when the variances were homogeneous or with Tamhane’s T2 and Dunnett's T3 post hoc tests when the variances were unequal. The homogeneity of variances was confirmed using Levene’s test. All tests were two-tailed. The results from in vitro experiments were determined from at least three biological replicates. The data are presented as the mean ± SEM. Differences were considered statistically significant at *P* < 0.05.

## Results

### Serum E_2_ declines as the gestational week increases in early pregnancy loss and decidual SGK1 is downregulated in macrophages of RPL

**Fig. 1:**
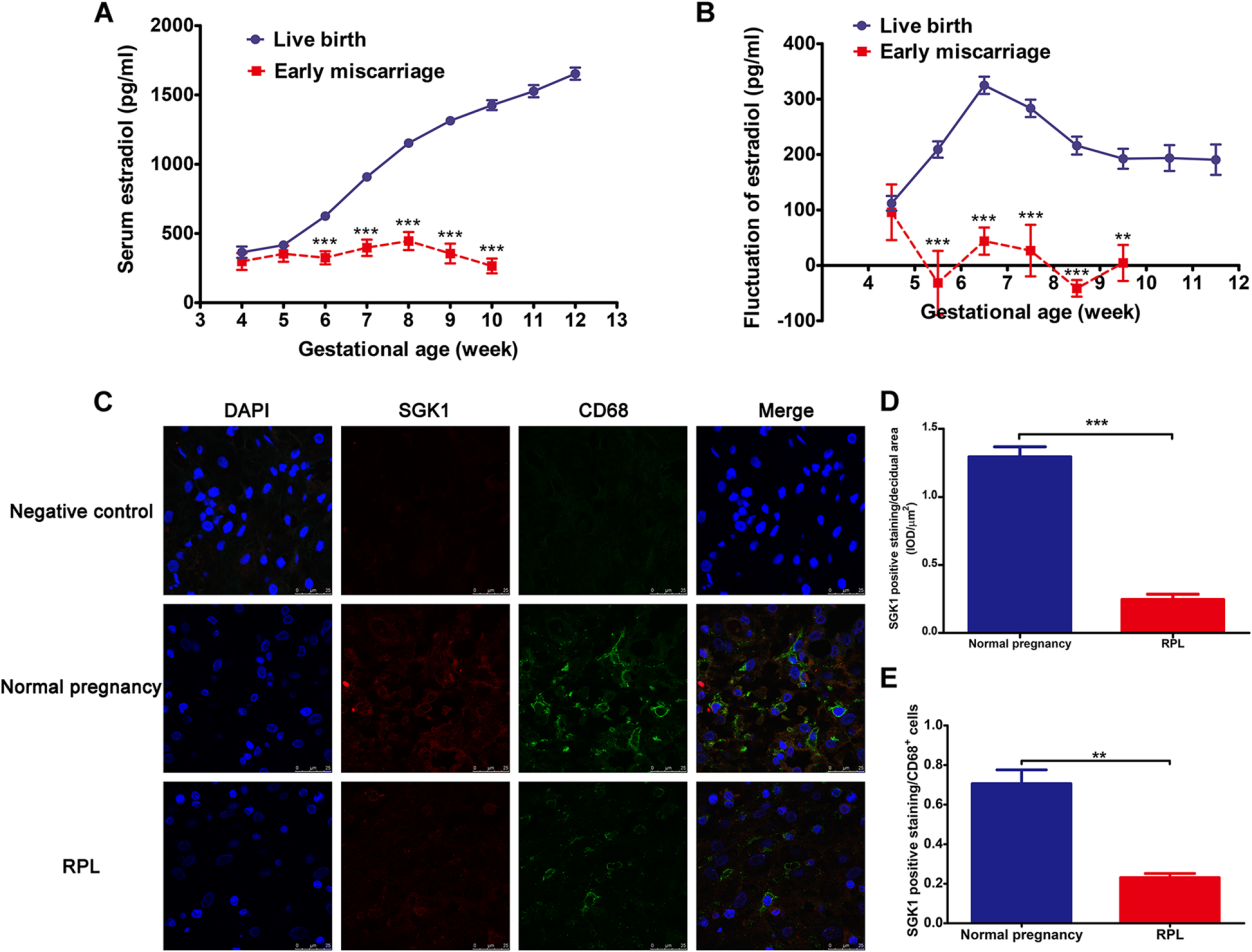
Serum E_2_ declines as gestational age increases in early miscarriage and SGK1 is downregulated in decidual macrophages of RPL. **A** The concentrations of serum E_2_ (pg/ml) during the 4^th^–12^th^ week of gestation in a study population with live birth (*n* = 448) or miscarriage (*n* = 68) in the first trimester. **B** The variation of serum E_2_ (pg/ml) with weeks during early pregnancy in women with a viable fetus or miscarriage. **C** Decidual tissue sections obtained from first trimester of gestation were double stained with anti-human SGK1 antibody (red) and anti-human CD68 antibody (green) using laser scanning confocal microscopy; nuclear DNA was stained with DAPI (blue). The yellow-orange color in the merged images indicates the colocalization of SGK1 and CD68^+^ macrophages in decidual tissue, *n* = 3, at 630 × magnification, scale bar 25 μm. **D** The ordinate represents the quantification of immunoreactive SGK1 levels/decidual area (μm^2^) acquired in (C). **E** The ordinate indicates the quantification of immunoreactive SGK1 staining in decidual CD68^+^ macrophages obtained from 1C. **P < 0.01, ***P < 0.001, contrasted to normal pregnancy group. E_2_, estradiol; SGK1, serum-glucocorticoid regulated kinase; RPL, recurrent pregnancy loss; CD68, CD68 molecule; DAPI, 4’,6-diamidino-2-phenylindole

Considering the importance of pregnancy-related hormones in pregnancy maintenance, we initially investigated the level of serum E_2_ in normal and compromised pregnancies. We found that the E_2_ concentrations were elevated as the gestational age (week) increased in early human pregnancy (Fig. 1A). However, from the 6^th^ week of gestation and later during early pregnancy, the levels of serum E_2_ were lower in the patients that suffered a miscarriage compared with those who experienced a live birth. Moreover, in the miscarriage group, we observed a lower increase in E_2_ concentrations compared with that in the live birth group, which further declined as gestational ages increased (Fig. 1B). This was consistent with our previous findings [37]. Estrogen is an essential steroid hormone for the coordinated uterine responses and sustained pregnancy [45]. Maternal serum E_2_ has been suggested as a promising biochemical marker to predict the outcome of threatened miscarriage [46]. Moreover, a lower level of serum E_2_ (Supplementary Table S2) was observed in women suffering from RPL than in those with a normal pregnancy. These data indicated that the early pregnancy loss was associated the lower E_2_ levels and a slower increment of serum E_2_.

Our previous research found that SGK1, which regulates some specific targets downstream of E_2_ [47] correlates with early threatened miscarriage [37]. Herein, we found that the expression of *SGK1* in the decidual tissue from the RPL group was decreased compared to that of the normal pregnancy group (Supplementary Table S2). Immunofluorescence labeling (Fig. 1C) showed that SGK1 was co-expressed with CD68, a surface marker for pan-macrophages, and was downregulated in decidual CD68^+^ macrophages from the RPL group compared with that from the normal pregnancy group (Fig. 1D and 1E). These results suggested a correlation between the compromised pregnancy in the first trimester of gestation and suppressed activation of SGK1 in decidual macrophages.

### E_2_ upregulates SGK1 activity via estrogen receptor β (ER β) in THP-1 monocyte-derived macrophages

**Fig. 2:**
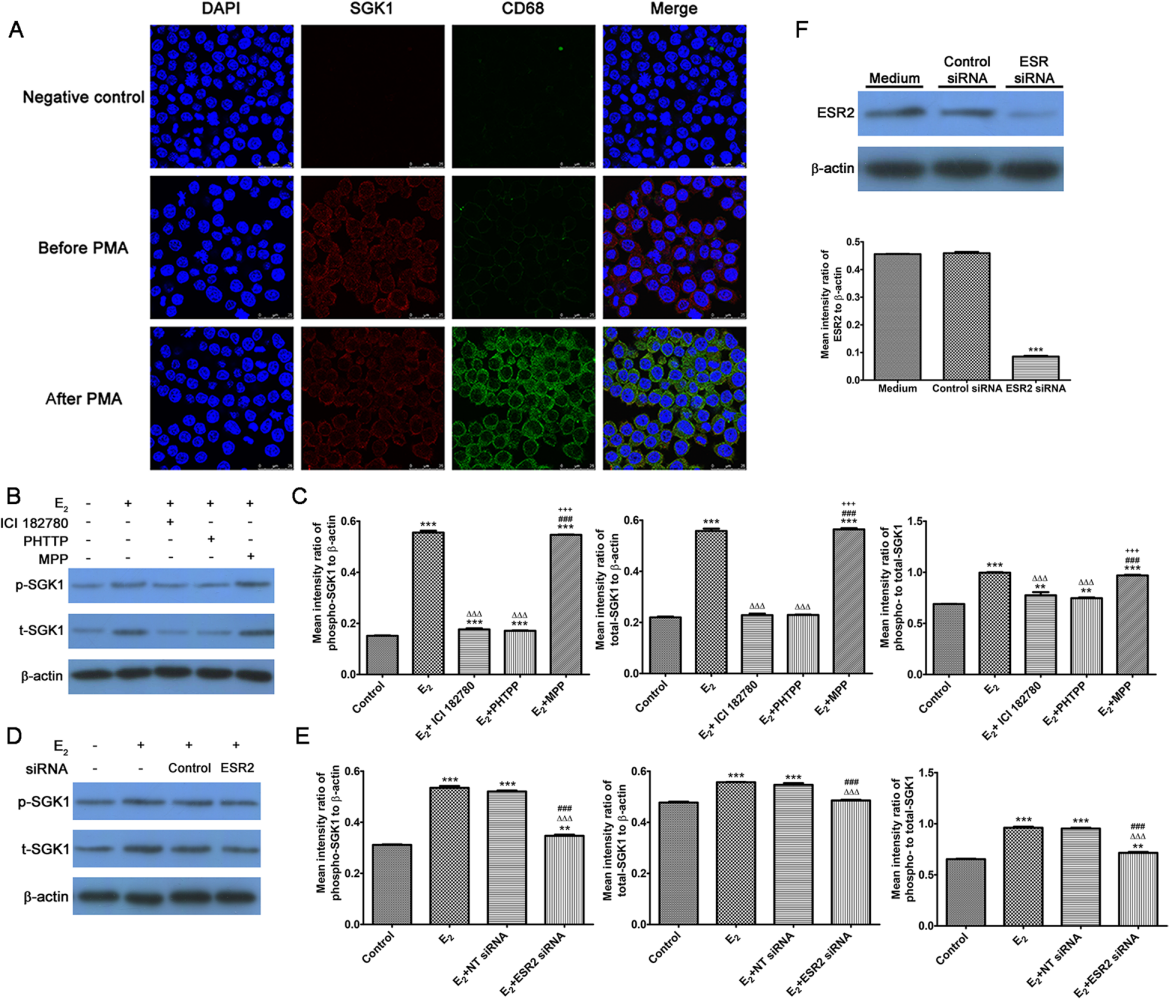
E_2_ upregulates SGK1 activity in THP-1 monocyte-derived macrophages via ERβ. **A** Representative immunofluorescence staining of THP-1 monocyte-derived macrophages before (resting phenotype, top), and after (differentiated phenotype, bottom) PMA incubation. The differentiation of macrophages was confirmed by staining with the antibodies against macrophage-specific CD68 (green) and with DAPI (blue) for nuclei. Final magnification: × 630, scale bar 25 μm. **B** Western blotting analysis of THP-1 monocyte-derived macrophages treated with E_2_ (10 nM) alone, E_2_ plus ER antagonist ICI182780 (1 μM), E_2_ plus ERα antagonist MPP (1 μM) and E_2_ plus ERβ antagonist PHTPP (1 μM) for 24 h. Blots were probed with antibodies to t-SGK1, p-SGK1, and β-actin. **C** Densitometric quantifications of the arithmetic mean (SEM) ratio of p-SGK1 (left), t-SGK1 (middle), and p/t-SGK1 protein (right) to β-actin in THP-1 monocyte-derived macrophages. Western blotting analysis (**D**) and densitometric quantifications (**E**) of THP-1 monocyte-derived macrophage lysates pretreated with an *ESR2* (encoding ERβ)-specific siRNA and non-targeting (NT) siRNA. Blots were probed with antibodies to p-SGK1 (left), t-SGK1 (middle), and p/t SGK1 protein (right) in THP-1 monocyte-derived macrophage lysates. β-actin was the loading control. **F** Blots were probed with antibodies to ESR2 and β-actin prepared from lysates of cell pretreated with NT siRNA or *ESR2* siRNA. Three independent samples were analyzed. Data are expressed as the arithmetic means ± SEM. **P < 0.01, ***P < 0.001, contrasted with control or medium group; ∆∆∆*P* < 0.001, contrasted with E_2_ group; ###*P* < 0.001, contrasted with the E_2_ + ICI 182780 or E_2_ + NT siRNA group; +  +  + *P* < 0.001, contrasted with the E_2_ + PHTPP group. E_2_, estradiol; SGK1, serum-glucocorticoid regulated kinase; ER, estrogen receptor; PMA, phorbol 12-myristate 13-acetate; CD68, CD68 molecule; DAPI, 4′,6-diamidino-2-phenylindole; NT, non-targeting; siRNA, small interfering RNA; SEM, Standard Error of the Mean

To mimic the TLR-mediated responses of maternal immunomodulation of the decidual macrophages, we used human THP-1 monocytes as a model cell line. Resting human THP-1 monocytes were differentiated into macrophages by incubation with PMA (Fig. 2A). Different concentrations and incubation times of PMA were tested, and we selected 120 ng/ml PMA incubation overnight followed by 24 h in control medium as the differentiation protocol.

In a previous study, we found that E_2_ could induce SGK1 expression and promote its phosphorylation in DSCs from early miscarriage [37]. Consequently, we examined the roles of estrogen receptor alpha (ERα) and ERβ in the regulation of SGK1 in THP-1 monocyte-derived macrophages. We found that E_2_ treatment significantly elevated total (t)-SGK1, phosphorylated (p)-SGK1, and p/t-SGK1 levels in THP-1 monocyte-derived macrophages (Fig. 2B and 2C), and these effects could be abrogated using the ER antagonist ICI182780 or the ERβ antagonist PHTTP, but not the ERα antagonist MPP. Moreover, siRNA-mediated *ESR2* knockdown showed that E_2_ upregulated the expression of SGK1 in THP-1 monocyte-derived macrophages (Fig. 2D–F). Therefore, our data showed that E_2_ upregulates the expression of SGK1 in THP-1 monocyte-derived macrophages via ERβ, which was consistent with the finding that ERβ regulates endometriotic cell survival through SGK1 activation [48].

### LPS/TLR4 signaling primes the pro-inflammatory Th1 immune profile of THP-1 monocyte-derived macrophages, and E_2_ modulates SGK1 activity via the PI3K pathway in LPS-stimulated macrophages

**Fig. 3:**
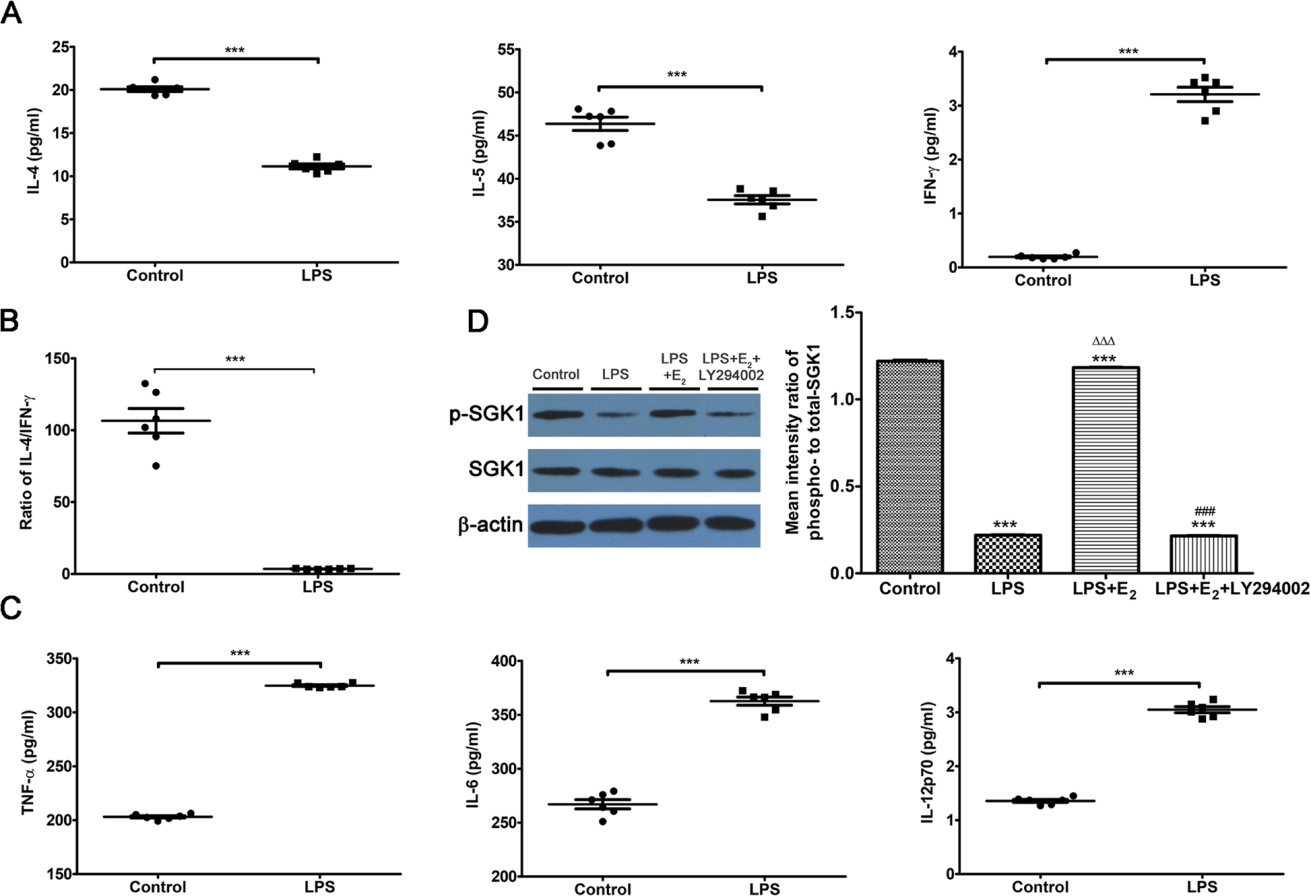
LPS/TLR4 promotes a pro-inflammatory Th1 immune profile, and E_2_ enhances SGK1 activity through PI3K in LPS-stimulated THP-1 macrophages. **A** Levels of IL4 and IL-5 (Th2 cytokines), and IFN-γ (Th1 cytokine) in the supernatants of THP-1-derived macrophages treated with or without LPS. **B** The ratio of IL-4/IFN-γ (Th2/Th1 cytokines) derived from the data in (**A**). **C** Cell-free supernatants were collected after 24 h of stimulation with (LPS group) or without (control group) the TLR4 ligand LPS derived from *Escherichia coli* 0111:B4, and LPS-triggered secretions of pro-inflammatory cytokines IL-12p70, TNF-α, and IL-6 of THP-1-derived macrophages were determined using a ProcartaPlex® Multiplex Immunoassay. **D** Western blot (left) of whole-cell lysates from LPS-stimulated macrophages pretreated with E_2_ in the presence and absence of PI3K inhibitor LY294002. Blots were probed with antibodies to t-SGK1, p-SGK1, and total β-actin. Densitometric quantification (right) of the arithmetic mean (SEM) ratio of phospho-to-total proteins for SGK1 in LPS-triggered macrophages pretreated with E_2_ in the presence and absence of LY294002. Three to six independent samples were analyzed. Data are presented as the arithmetic means ± SEM. ***P < 0.001, contrasted with the control group; ∆∆∆*P* < 0.001, contrasted with the LPS group; ###*P* < .001, contrasted with the LPS + E_2_ group. LPS, lipopolysaccharide; TLR4, Toll-like receptor; Th, T helper; E_2_, estradiol; SGK1, serum-glucocorticoid regulated kinase; PI3K, phosphoinositide 3-kinase; IL, interleukin; IFN-γ, interferon gamma; TNF, tumor necrosis factor; p-, phospho-; SEM, Standard Error of the Mean

LPS/TLR4 signaling is associated with miscarriage, and is used to induce models of abortion [49]. Here, we used LPS-induced macrophages to mimic the immune response of decidual macrophages at the maternal–fetal interface of RPL. Notably, the accumulation of Th2 cytokines, such as IL-4 and IL-5, was significantly diminished, while the level of the Th1 cytokine IFN-γ was increased in the supernatants of THP-1 monocyte-derived macrophages after LPS stimulation compared with that in the control cells (Fig. 3A). Moreover, the ratio of Th2/Th1 cytokines (IL-4/IFN-γ) was decreased in LPS-triggered macrophages compared with that of the control cells (Fig. 3B). Subsequently, we assessed the generation of pro-inflammatory cytokines, such as IL-12p70, TNF-α, and IL-6, in response to stimulation with the TLR4 agonist LPS. Increased secretions of pro-inflammatory cytokines, characterized by increased TNF-α, IL-6, and IL-12p70 productions were observed in LPS-treated THP-1 macrophages compared with that in the control resting macrophages (Fig. 3C). Therefore, when exposed to LPS treatment, our results show a polarization towards pro-inflammatory Th1 potentiation in THP-1-derived macrophages, which would be deleterious to gestation maintenance.

E_2_ treatment notably increased phosphorylation level of SGK1 in THP-1 monocyte-derived macrophages. Studies have demonstrated that the PI3K signaling pathway is an important activator of SGK1; PI3K-mediated activation of SGK1 upon various stimuli has been observed in different cells [50]. Previous work reported a connection between E_2_ activities and SGK1 phosphorylation in LPS-induced acute lung injury [51]. Therefore, we assessed the molecular alterations in THP-1 monocyte-derived macrophages upon LPS stimulation. Compared with that in resting macrophages, phosphorylation of SGK1 was reduced in THP-1-derived macrophages pretreated with LPS (Fig. 3D). However, this reduction was abrogated by the presentation of E_2_. Then, we applied the PI3K inhibitor LY294002 to the LPS-stimulated THP-1 macrophages incubated with E_2_. Western blotting analysis showed that the blockade of the PI3K signaling pathway abrogated the phosphorylation of E_2_-activated SGK1 in LPS-stimulated THP-1 macrophages. These findings confirmed that the previously identified involvement of E_2_ in the activation of SGK1 via the PI3K pathway also occurred in LPS-stimulated macrophages [52].

### Specific SGK1 inhibition blocks E_2_-induced M2 macrophage transition and Th2 immune shift in THP-1 monocyte-derived macrophages

**Fig. 4:**
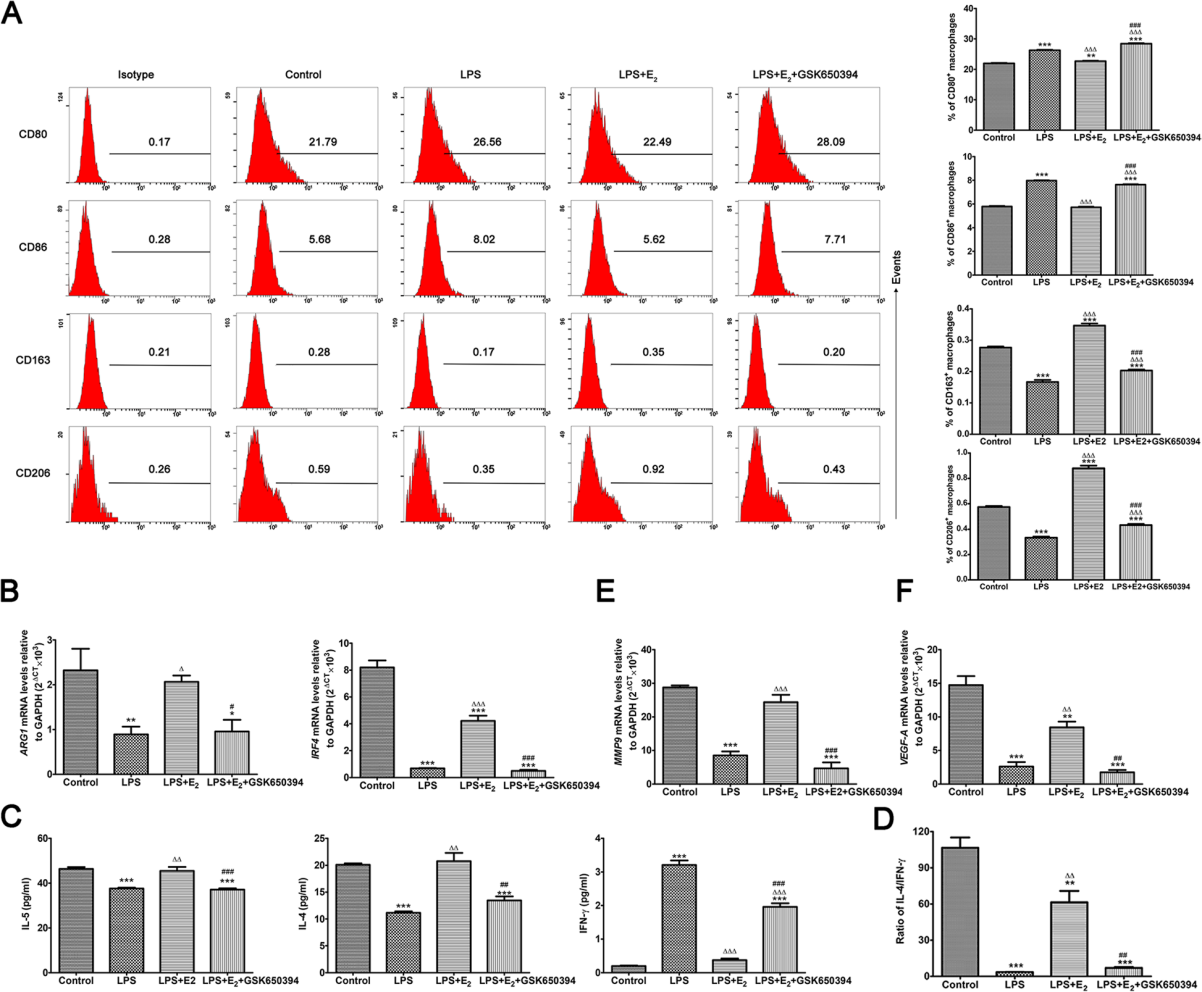
SGK1 inhibition blocks E_2_-triggered M2 macrophage transition and Th2 immune responses in THP-1-derived macrophages. **A** Flow cytometry analysis and quantification of the expression of M2 markers CD206 and CD163, as well as M1 markers CD80 and CD86, in LPS-stimulated macrophages pretreated with E_2_ in the presence or absence of the SGK1 inhibitor GSK650394. The plot show one representative flow cytometric analysis. **B** Transcript levels of *ARG1* and *IRF4* (differentiated M2 markers) were assessed using qRT-PCR in LPS-trigged macrophages pretreated with E_2_ in the presence or absence of GSK650394. **C** Concentrations of IL4 and IL-5 (Th2 cytokines), and IFN-γ (Th1 cytokine) in the supernatants of LPS-stimulated macrophages pretreated with E_2_ in the presence or absence of GSK650394. **D** Quantification of the arithmetic mean (SEM) ratio of IL-4/IFN-γ (Th2/Th1 cytokines) obtained from data presented (C). qRT-PCR analysis showing the mRNA levels of *MMP9* (**E**), and *VEGFA* (**F**) in the presence or absence of GSK650394 in LPS-stimulated macrophages pretreated with E_2_. *GAPDH* served as the internal control. Data are presented as the arithmetic means ± SEM for three individual experiments. *P < 0.05, **P < 0.01, ***P < 0.001, contrasted with the control group; ∆*P* < 0.05, ∆∆*P* < 0.01, ∆∆∆*P* < 0.001, contrasted with the LPS group; #*P* < 0.05, ##*P* < 0.01, ###*P* < 0.001, contrasted with the LPS + E_2_ group. SGK1, serum-glucocorticoid regulated kinase; E_2_, estradiol; ARG1, arginase 1; IRF4, immune regulatory factor 4; LPS, lipopolysaccharide; IL, interleukin; IFN-γ, interferon gamma; MMP9, matrix metalloproteinase 9; VEGF-A, vascular endothelial growth factor A; GAPDH, glyceraldehyde-3-phosphate dehydrogenase; SEM, Standard Error of the Mean

As described above, LPS/TLR4 signaling induced a Th1 immune response of macrophages. Intriguingly, E_2_ supplementation reversed these effects of LPS treatment. Pre-incubation with 10 nM E_2_ restored the expressions of CD163 as well as that of CD206 in TLR4-activated macrophages. Interestingly, as the downstream target of E_2_, SGK1 has been implicated in macrophage differentiation [53]. Accordingly, consistent with the influence of E_2_ on the polarization of macrophages, pharmacological inhibition of SGK1 activation released E_2_-blocked expression of CD80 and CD86 (M1 markers) in LPS-stimulated macrophages, whereas it decreased the expression of M2 markers CD163 and CD206 (Fig. 4A). Subsequently, we examined the expression levels of genes related to the M2 phenotype. E_2_ supplementation substantially rescued the TLR4-mediated reduction of both *IRF4* (encoding immune regulatory factor 4) and *ARG1* (encoding arginase 1) transcripts in THP-1 monocyte-derived macrophages (Fig. 4B). *IRF4* has been also implicated in Th2 cytokine accumulation at the maternal–fetal interface [54]; therefore, we next determined the Th2 immune reactions upon E_2_ stimulation in THP-1-derived macrophages. E_2_ pretreatment promoted the secretion of Th2 cytokines, such as IL-4 and IL-5, whereas it inhibited the secretion of Th1 cytokine IFN-γ in LPS-activated macrophages (Fig. 4C). Blockade of SGK1 significantly downregulated the production of Th2 cytokines by macrophages when compared with those pretreated with E_2_, as evidenced by the reduced ratio of IL-4/IFN-γ (Th2/Th1 cytokines, Fig. 4D). Studies have reported that SGK1 selectively regulates the production of Th1 and Th2 cytokines in CD4^+^ T cells [55]. Therefore, our data also suggested that SGK1 activation promotes Th2 cytokine production in LPS-stimulated macrophages, which is beneficial to a normal healthy pregnancy. Furthermore, we examined SGK1-dependent gene expression of *MMP9* (encoding matrix metalloproteinase 9) and *VEGFA* (encoding vascular endothelial growth factor-A), two transcripts expressed in response to Th2 cytokines [56, 57]. To this end, THP-1-derived macrophages were supplemented with E_2_ or pre-incubated with the SGK1 inhibitor GSK650394 (10 μM) after stimulation with LPS. As illustrated in Fig. 4E and Fig. 4F, E_2_ supplementation substantially reversed the TLR4-mediated reduction of in *MMP9* and *VEGFA* expression, whereas blockade of SGK1 ablated these effects. These findings suggested that E_2_-triggered SGK1 activation in macrophages contributes to M2-Th2 polarization at the maternal–fetal interface, ensuring an immunotolerant intrauterine environment that supports successful gestation maintenance.

### E_2_-sensitive activation of SGK1 negatively regulates NF-κB activity to compromise macrophage secretion of pro-inflammatory cytokines

**Fig. 5:**
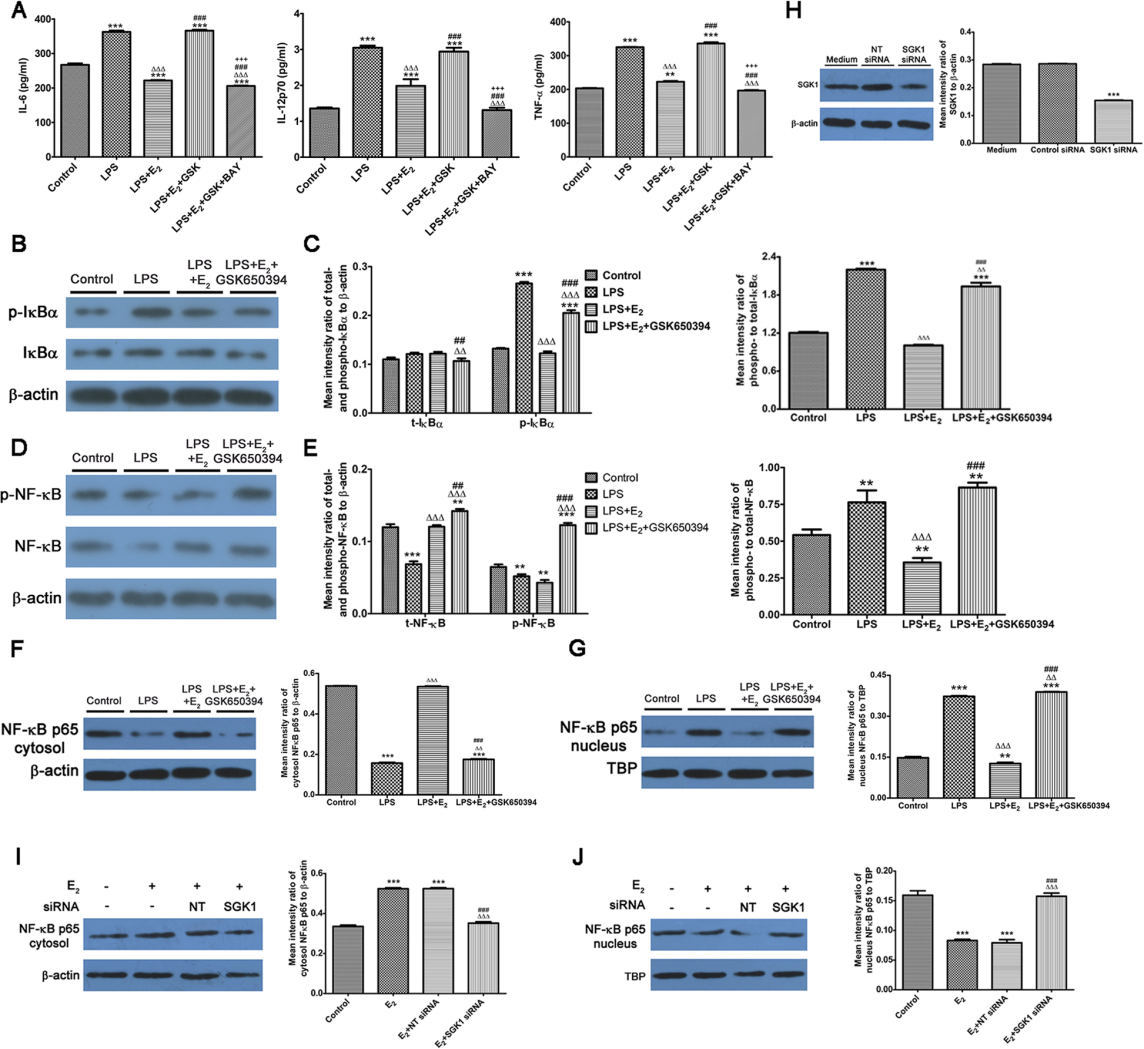
E_2_-activated SGK1 negatively regulates NF-κB to compromise the secretion of pro-inflammatory cytokines by macrophages. THP-1 monocyte-derived macrophages stimulated by LPS were pretreated with E_2_ in the presence or absence of GSK650394 (SGK1 inhibitor) and BAY11-7082 (NF-κB inhibitor). Whole-cell lysates, cytosol and nucleus lysates, and cell-free supernatants were collected to assess the cytokines levels and NF-κB activities, respectively. **A** TLR4-mediated production of pro-inflammatory cytokines TNF-α, IL-6, and IL-12p70 of THP-1 monocyte-derived macrophages pretreated with E_2_ in the presence or absence of GSK650394 and BAY11-7082 was determined using the ProcartaPlex® Multiplex Immunoassay. **B** Western blot of whole cell lysates probed for p-IκBα, t-IκBα, and total β-actin. **C** The ratio of phospho-to-total proteins for IκBα (right) and relative protein levels of p-IκBα and t-IκBα to β-actin (left) were determined using densitometry. **D** Blots probed with antibodies for p-NF-κB, t-NF-κB, and total β-actin. **E** The ratio of phospho-to-total proteins for NFκB (right), and the relative protein levels of p-NF-κB and t-NF-κB to β-actin (left) were determined using densitometry. **F** Cytosol lysates probed for cytosolic NF-κB p65 and β-actin (left), and densitometric quantification of the ratio of the cytosolic level of NFκB p65 to β-actin (right). **G** Nuclear lysates probed for nuclear NF-κB p65 and TBP (left), and the densitometric quantification of the ratio of the nuclear level of NFκB p65 to TBP (right). Blots probed for SGK1 (**H**), cytosolic (**I**) and nuclear (**J**) NF-κB p65 of lysates of macrophages following non-targeting (NT) or *SGK1*-specific RNA silencing and then stimulated with E_2_. Data are the arithmetic means ± SEM for three biological replicates. **P < 0.01, ***P < 0.001, contrasted with the control or medium group; ∆∆*P* < 0.01, ∆∆∆*P* < 0.001, contrasted with the LPS group or E_2_ group; ###*P* < 0.001, contrasted with the LPS + E_2_ group or E_2_ + NT siRNA group; +  +  + *P* < 0.001, contrasted with the LPS + E_2_ + GSK650394 group. E_2_, estradiol; SGK1, serum-glucocorticoid regulated kinase; NF-κB; nuclear factor kappa B; TNF, tumor necrosis factor; IL, interleukin; p-, phospho-; t-, total-; IκBα, inhibitor of nuclear factor kappa-B kinase subunit alpha; TBP, TATA binding protein; SEM, Standard Error of the Mean; GSK, GSK650394; BAY, BAY11-7082

To probe the molecular mechanisms by which SGK1 inhibition might lead to pro-inflammatory responses, we next explored the NF-κB pathway, which lies downstream of PI3K/SGK1 signaling. Our data demonstrated that blockade of NF-κB activity using the inhibitor BAY11-7082 counteracted the enhanced generation of pro-inflammatory cytokines IL-6, IL-12p70, and TNF-α by LPS-stimulated macrophages, and also offset the influence of SGK1 inhibition (Fig. 5A). Subsequently, we performed western blotting analysis of members of the NF-κB signaling pathway. LPS stimulation led to elevated levels of phosphorylated NF-κB inhibitor alpha (IκBα) (Fig. 5B and 5C) and increased ratio of phosphorylated NF-κB (Fig. 5D and 5E) compared with those in the control macrophages. Consistently, further investigation of TLR4-induced protein abundance of NF-κB p65 in the nucleus confirmed that the nuclear translocation of NF-κB p65 was upregulated following LPS stimulation in THP-1 monocyte-derived macrophages (Fig. 5F and 5G). E_2_ incubation antagonized the TLR4-mediated effects to block the increased in phosphorylated IκBα and NF-κB. Western blotting showed that the nuclear translocation of NF-κB p65 was also decreased after E_2_ treatment. By contrast, inhibition of SGK1 resulted in upregulated levels of phosphorylated IκBα and NF-κB, and the nuclear translocation of NF-κB p65, after LPS stimulation in THP-1 monocyte-derived macrophages, a result that was in partial agreement with data from a previous work [58]. To exclude the possible nonspecific impact of GSK650394, we employed an SGK1 siRNA, which confirmed that SGK1 negatively regulated NF-κB nuclear translocation and subsequent activation in LPS-triggered macrophages upon E_2_ stimulation (Fig. 5H-J).

Then, we investigated the functional effect of E_2_-SGK1-NF-κB on LPS-induced accumulation of intracellular cytokines. E_2_ pre-incubation with THP-1-derived macrophages markedly suppressed TLR4-mediated elevation secretion of pro-inflammatory IL-12p70, TNF-α, and IL-6, which are involved in pregnancy loss. Conversely, pharmacological inhibition of E_2_-dependent SGK1 induction abrogated these protective effects of E_2_ on LPS-triggered macrophages, while NF-κB blockade dramatically reduced these excessive inflammatory responses, which are deleterious tohealthy gestation (Fig. 5A). These findings suggested that E_2_-activated SGK1 reduces the generation of pro-inflammatory cytokines by LPS-stimulated macrophages via inhibiting the nuclear translocation and subsequent activation of NF-κB.

### E_2_ promotes SGK1 activation and suppresses nuclear translocation of NF-κB in uterine macrophages of ovariectomized mice

**Fig. 6:**
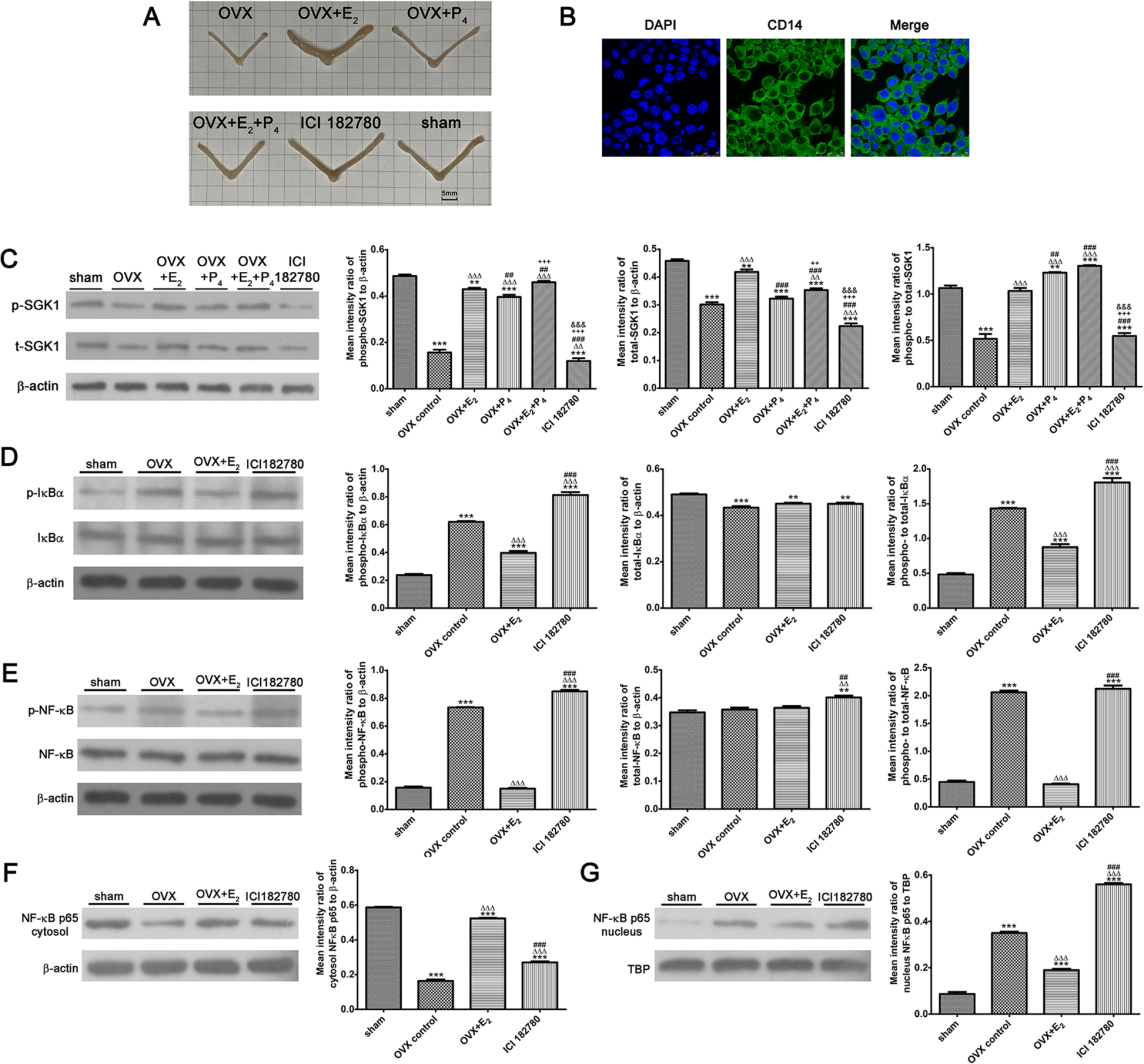
E_2_ activates SGK1 and promotes the cytosol location of NF-κB in uterine macrophages of ovariectomized (OVX) mice. Eight to ten week old female ICR mice were divided randomly into a sham group and five experimental groups (*n* = 10). The mice in the five experimental groups were ovariectomized before treatment with different hormone combinations. **A** The uterine morphologies in mice injected with steroid hormones or ICI 182780. **B** Uterine macrophages were extracted from the uteruses of the female mice that received either vehicle (corn oil), E_2_, P_4_, E_2_ + P_4_, or the ER antagonist ICI 182780 after OVX. Final magnification: × 630, scale bar 25 μm. (C-G) Western blotting analysis in uterine macrophages of mice treated with E_2_ (100 ng) alone, P_4_ (1 mg) alone, E_2_ (100 ng) plus P_4_ (1 mg), ER antagonist ICI182780 (100 μg), or corn oil (100 μL) after OVX and in the sham group. **C** Blots were probed with antibodies to t-SGK1, p-SGK1, and β-actin. **D** Western blotting analysis of p-IκBα, t-IκBα, and total β-actin. **E** Blots probed for p-NF-κB, t-NF-κB, total β-actin. **F** Cytosol lysates of uterine macrophages probed for cytosolic NF-κB p65 and β-actin. **G** Nucleus lysates probed for nuclear NF-κB p65 and TBP in uterine macrophages. Data are the arithmetic means ± SEM for three biological replicates. **P < 0.01, ***P < 0.001, contrasted with the sham group; ∆∆*P* < 0.01, ∆∆∆*P* < 0.001, contrasted with the OVX control group; ##*P* < 0.01, ###*P* < 0.001, contrasted with the OVX + E_2_ group; +  + *P* < 0.01, +  +  + *P* < 0.001, contrasted with the OVX + P_4_ group; &&&*P* < 0.001, contrasted with the OVX + E_2_ + P_4_ group. E_2_, estradiol; SGK1, serum-glucocorticoid regulated kinase; OVX, ovariectomized; P_4_, progesterone; ER, estrogen receptor; p-, phospho-; t-, total-; NF-κB; nuclear factor kappa B; IκBα, inhibitor of nuclear factor kappa-B kinase subunit alpha; TBP, TATA binding protein; SEM, Standard Error of the Mean

To further verify the mechanisms underlying the regulation of SGK1 activation by E_2_ in macrophages, we investigated the expression and phosphorylation of SGK1 in the uterine macrophages of OVX mice following hormone treatment. The OVX mouse model has been widely used in research to examine the effects of ovarian hormones on the endometrium [28, 59]. In the in vivo experiments, macrophages were extracted from the uterine tissues of female mice that received either vehicle (corn oil), E_2_, P_4_, E_2_ + P_4_, or the ER antagonist ICI 182780 for 24 or 72 h after OVX (Fig. 6A and 6B). Western blotting analysis showed that SGK1 levels were very low in the uterine macrophages of OVX mice and that E_2_ supplementation increased the level of SGK1 (Fig. 6C). While E_2_ increased the phosphorylation of SGK1 in the uterine macrophages of OVX mice, ICI 182780 significantly inhibited this E_2_-induced phosphorylation. Administration of E_2_ significantly reduced the levels of phosphorylated IκBα (Fig. 6D) and NF-κB (Fig. 6E), and these repressive effects were rescued using the ER antagonist. Furthermore, western blotting analyses revealed that the level of nuclear NF-κB was markedly reduced in the uterine macrophages of OVX mice injected with E_2_ (Fig. 6G). Contrastingly, cytosolic NF-κB levels were markedly increased in uterine macrophages from OVX mice injected with E_2_ (Fig. 6F). Interestingly, this induction of cytosolic NF-κB was also blocked by ICI 182780. Taken together, these data suggested that E_2_ increases the phosphorylation of SGK1 and represses the nuclear translocation of NF-κB in uterine macrophages in vivo.

## Discussion

In the present study, we investigated whether E_2_-activated SGK1 is involved in macrophage polarization at the maternal–fetal interface. Our findings demonstrated that SGK1 activation induced by E_2_ is an important factor in defining decidual macrophage polarization. SGK1 activation increases the characteristic gene expressions of M2 macrophages and enhances the anti-inflammatory Th2 cytokine secretions of decidual macrophages in the intrauterine immune microenvironment. Deficiency of SGK1 activity promoted the production of pro-inflammatory Th1 cytokines and reduced M2-specific gene expression, indicating that E_2_-activated SGK1 is important for the polarization of uterine macrophages. We also found that SGK1 regulates NF-κB to suppress the generation of excessive pro-inflammatory cytokines by driving its cytosolic translocation. In addition, we demonstrated this functional effect of E_2_-SGK1-NF-κB in uterine macrophages from OVX mice. Therefore, E_2_-activatied SGK1 is pivotal for macrophage polarization at the maternal–fetal interface, which could be an interventional target to manage recurrent implantation failure and RPL.

Successful establishment and maintenance of pregnancy depends heavily on the generation and maintenance of maternal–fetal immune tolerance [2]. Interruption of this dynamic immune balance at the maternal–fetal interface is related to pregnancy-related complications [43]. It has been recognized that decidual macrophages participate in intrauterine immune modulation during pregnancy [10]. The appropriate polarization state of decidual macrophages is closely related to their functions, which are essential to maintain the normal immune microenvironment during pregnancy; otherwise, it can result in adverse pregnancy outcomes, including RPL [60]. Decidual macrophages exhibiting the M2 phenotype are one of the important immune characteristics of normal pregnancy [61]. Enhanced M1 differentiation, characterized by increased expression of pro-inflammatory cytokines, is associated with RPL [43]. The exact mechanisms involved in the polarization of decidual macrophages and the roles of these events during early pregnancy are not well understood.

We observed that decidual macrophages in women with normal pregnancy exhibited higher expression of SGK1 than that of women with RPL. As a highly conserved kinase, SGK1 has been detected in the human and mouse decidual, as well as human endometrial stromal cells (HESCs), and HEK293T and Ishikawa cell lines [34, 62]. A study reported that compromised pregnancies have a substantially lower expression of decidual SGK1 than that of normal pregnancies [62]. Our previous results also showed less expression of decidual SGK1 at the maternal–fetal interface of women suffering from early spontaneous abortion [37]. The results of the present study confirmed this observation in the decidua from patients with RPL. Moreover, co-localization of SGK1 and macrophages was observed at the maternal–fetal interface of the RPL samples. Importantly, we found that lower levels of serum E_2_ correlated with the down-regulation of SGK1 in decidual tissue from the RPL group. Interestingly, both E_2_ and SGK1 have been reported to influence the phenotypes of macrophages [32]. Therefore, we speculated that E_2_-SGK1 signaling could affect the functions of decidual macrophages, contributing to maintaining homeostasis and reproductive success.

E_2_ regulates innate immune function and attenuates the generation of pro-inflammatory cytokines, such as IL-6 [63]. Intriguingly, E_2_ displays both pro- and anti-inflammatory effects on the immune system. E_2_ promotes the initial inflammatory mediator, LPS/TLR4, signaling to directly stimulate peritoneal macrophages to secrete TNF-α and IL-6 in the internal milieu of pelvic inflammation within endometriosis [64]. In contrast, the activation of the intracellular ER by E_2_ incubation in a mouse RAW 264.7 cell line shortened the pro-inflammatory phase and accelerated the resolution of inflammation, favoring the progression of macrophages toward the IL-10-dependent acquired deactivation or the M2c phenotype, which is responsible for the restoration of tissue homeostasis by immunomodulation, angiogenesis and tissue remodeling [65]. Our present findings confirmed this conclusion in LPS-stimulated macrophages. We demonstrated that E_2_ pretreatment limited the LPS-triggered release of pro-inflammatory cytokines IL-6, IL-12p70, and TNF-α in THP-1 monocyte-derived macrophages. We utilized THP-1 monocyte-derived macrophages as a model cell line to mimic the differentiation of decidual macrophages at the maternal–fetal interface during early pregnancy of RPL because they can be readily induced into terminal phenotypes. We also showed that LPS pre-incubation reduced the production of Th2-type cytokines, such as IL-4 and IL-5, whereas it increased Th1-type cytokines secretion, such as IFN-γ, in THP-1 monocyte-derived macrophages. Meanwhile, E_2_ pre-incubation reversed these Th1 immune responses to LPS/TLR4 in the THP-1 monocyte-derived macrophages. Meanwhile, E_2_ pretreatment restored the TLR4-suppressed expression of M2 markers CD163 and CD206, promoting M2 polarization of THP-1 macrophages, as evidenced by the significant increment in *ARG1* and *IFR4* transcripts. Taken together, our data demonstrated that E_2_ incubation relieves uncontrolled activation of TLR4 signaling by skewing towards an anti-inflammatory cytokine profile at the maternal–fetal interface, leading to M2 macrophages and Th2 immune responses, which benefit normal pregnancy.

Macrophages enriched at the maternal–fetal interface are biased toward the immunosuppressive M2 subtype [66]. This polarization is implicated in several important biological processes during the pregnancy such as the immune tolerance of semi-allogeneic fetuses, tissue remodeling, host protection, resolution of inflammation, and securing a homeostatic intrauterine microenvironment [13]. Our data demonstrated that E_2_-sensitive SGK1 primed macrophage polarization toward the M2 subtype. M2 macrophages display an anti-inflammatory phenotype, which is closely associated with increased Th2 cytokines, whereas M1 macrophages correlate with pro-inflammatory immune responses with decreased Th2 cytokine levels [10]. In support of this, we also found that SGK1 inhibition abrogated the E_2_-induced increment of Th2 cytokines, as well as the reduction of Th1 cytokines in LPS-stimulated macrophages. There is increasing evidence for novel roles of SGK1 in T helper cell induction and differentiation [55]. Studies have shown that T helper type 17 (Th17) and regulatory T cells (Treg cells) adapt their function in a SGK1-dependent manner [33, 67]. These studies established the essential roles of SGK1 in the homeostasis of immune responses. Consistently, we found that SGK1 inhibition reduced the E_2_-stimulated expression of *IRF4*, which is also involved in Th2 polarization at the maternal–fetal interface in LPS-triggered macrophages [54]. Moreover, we showed that E_2_ pretreatment in THP-1 monocyte-derived macrophages substantially recovered TLR4-mediated transcriptional reductions of *MMP9* and *VEGF-A*, two genes involved in embryo implantation and RPL [68, 69]. Being heavily implicated in tissue remodeling and vasculogenesis, MMPs and VEGF are particularly important gestation regulators, because extracellular matrix degradation and neo-angiogenesis are required for the migration of macrophages into the stromal tissue and subsequent decidualization, which are prerequisites for cytotrophoblast invasion and gestation maintenance [62]. In our in vitro study, SGK1 inhibition abrogated E_2_-induced increment of *MMP9* and *VEGFA* expression in LPS-stimulated THP-1 macrophages, implying a promising role of E_2_-activated SGK1 in triggering Th2 immune reactions of macrophages to promote tissue remodeling and vasculogenesis in early gestation. These observations suggested that the E_2_-sensitive activation of SGK1 negatively regulates TLR4-mediated innate immune responses and participates in the skewing towards an anti-inflammatory Th2 responses, which could also facilitate decidual remodeling and vasculogenesis at the maternal–fetal interface by inducing M2 macrophages during early pregnancy.

The present study shed further light on the intricate molecular mechanism involved in the amplification of pro-inflammatory responses in response to E_2_-SGK1 inhibition in macrophages. We found that E_2_ induced the expression and activation of SGK1 through ER in uterine macrophages from OVX mice. In THP-1 monocyte-derived macrophages, we found that E_2_ augmented the level of phosphorylated SGK1, meanwhile an ERβ antagonist, but not an ERα antagonist, abolished these effects. It has been reported that *ESR1* (encoding ERα) affects the activities of SGK1 through the PI3K pathway in epithelial cells [52]. Recently, the *ESR2/SGK1* axis has been considered as a genetic combination in research into the genetic background of salt sensitivity hypertension concentration [70]. Herein, our data showed that E_2_ upregulates SGK1 activation via ERβ in macrophages. A recent study showed that E_2_ can activate SGK1 through the PI3K pathway [52]. In line with these previous reports, we showed that E_2_ pretreatment activated the downstream target SGK1 via PI3K signaling in LPS-challenged THP-1 macrophages: A PI3K inhibitor substantially diminished E_2_-induced phosphorylation of SGK1. Furthermore, SGK1 inhibition dramatically offset the effects of E_2_ supplementation on LPS-stimulated THP-1 macrophages, resulting in a shift toward to M1 macrophages. Therefore, we speculated that E_2_ activates SGK1 via the PI3K signaling pathway, which might also affect the differentiation of macrophages, ultimately contributing to immune tolerance at the maternal–fetal interface.

In the present study, our observations revealed that E_2_-activated SGK1 negatively regulated LPS-induced pro-inflammatory cytokines secretion by downregulating the activation of the transcriptional regulator NF-κB. Evidence shows that NF-κB is closely related to the development of pregnancy and has been reported to be involved in the apoptosis of endometrial epithelial cells to induce an inflammatory response, thereby reducing uterus receptivity via the TLR4/NF-κB/IL-6/VEGF pathway [71]. TLR4-NF-κB signaling is also activated in macrophages at the maternal–fetal interface, which could activate pyroptosis to cause aseptic inflammation, leading to the development of unexplained recurrent spontaneous abortion [72]. NF-κB is activated in response to E_2_ treatment in trophoblastic cells through ERα, because E_2_ stimulation significantly increased the expression of NF-κB p65 and phosphorylation of the inhibitory protein, IκBα, leading to the translocation of NF-κB p65 into the nucleus [73]. With respect to SGK1-dependent NF-κB signaling, SGK1 has been reported to promote the migration of macrophages by phosphorylating IκB kinase (IKKα/β), thereby facilitating IκBα phosphorylation and degradation, leading to the nuclear translocation of NF-κB p65 and subsequent NF-κB cascade activation [74, 75]. Pharmacological inhibition or silencing of IKK abrogated these inductive effects of SGK1 on the transcriptional activities of NF-κB [76]. Our findings demonstrated that activation of SGK1 upon E_2_ stimulation downregulated NF-κB-dependent transcriptional activities, while SGK1 blockade or knockdown reversed the inhibitory effects of E_2_ pretreatment on NF-κB activities in LPS-stimulated THP-1 macrophages. Moreover, we found that activated SGK1 regulated the translocation of NF-κB in uterine macrophages from OVX mice. Therefore, our data suggested that E_2_-activated SGK1 inactivates the NF-κB pathway in LPS-stimulated macrophages to attenuate the secretion of pro-inflammatory cytokines that are involved in pregnancy loss.

Additionally, we observed sluggish increment of the serum E_2_ concentration or even a decreased level of serum E_2_ from the 4^th^ to the 12^th^ week of gestation in women whose pregnancy ended in spontaneous miscarriages compared with those whose pregnancy ended with live birth. This difference became more obvious from the 6^th^ week of pregnancy. The time point of this difference is consistent with the key period of embryonic development from the 5^th^ week to the 6^th^ week of pregnancy. E_2_ is involved in the dramatic changes of the uterus throughout the entire human pregnancy, such as trophoblast invasion, allogeneic embryo implantation, and placental angiogenesis [30, 77]. We found that women suffering from RPL had lower level of serum E_2_ than that of women with a normal pregnancy. Since E_2_ regulates the expression of progesterone receptor (PGR) [78], and we also found that E_2_ up-regulates the expression of PGR, and P_4_ upregulated the expression of SGK1 in THP-1 cells. (Supplementary Figure S1). However, our data showed that the levels of serum P_4_ in early spontaneous miscarriage were similar to those of normal pregnancy, denoting a weak correlation between this serum biochemical marker and early miscarriage, as the majority of patients suffering from threatened miscarriage use progesterone supplementation. These results indicated that the level and increment of maternal serum E_2_ during early pregnancy could be regarded as a promising biochemical marker to predict the tendency for RPL.

## Conclusions

**Fig. 7:**
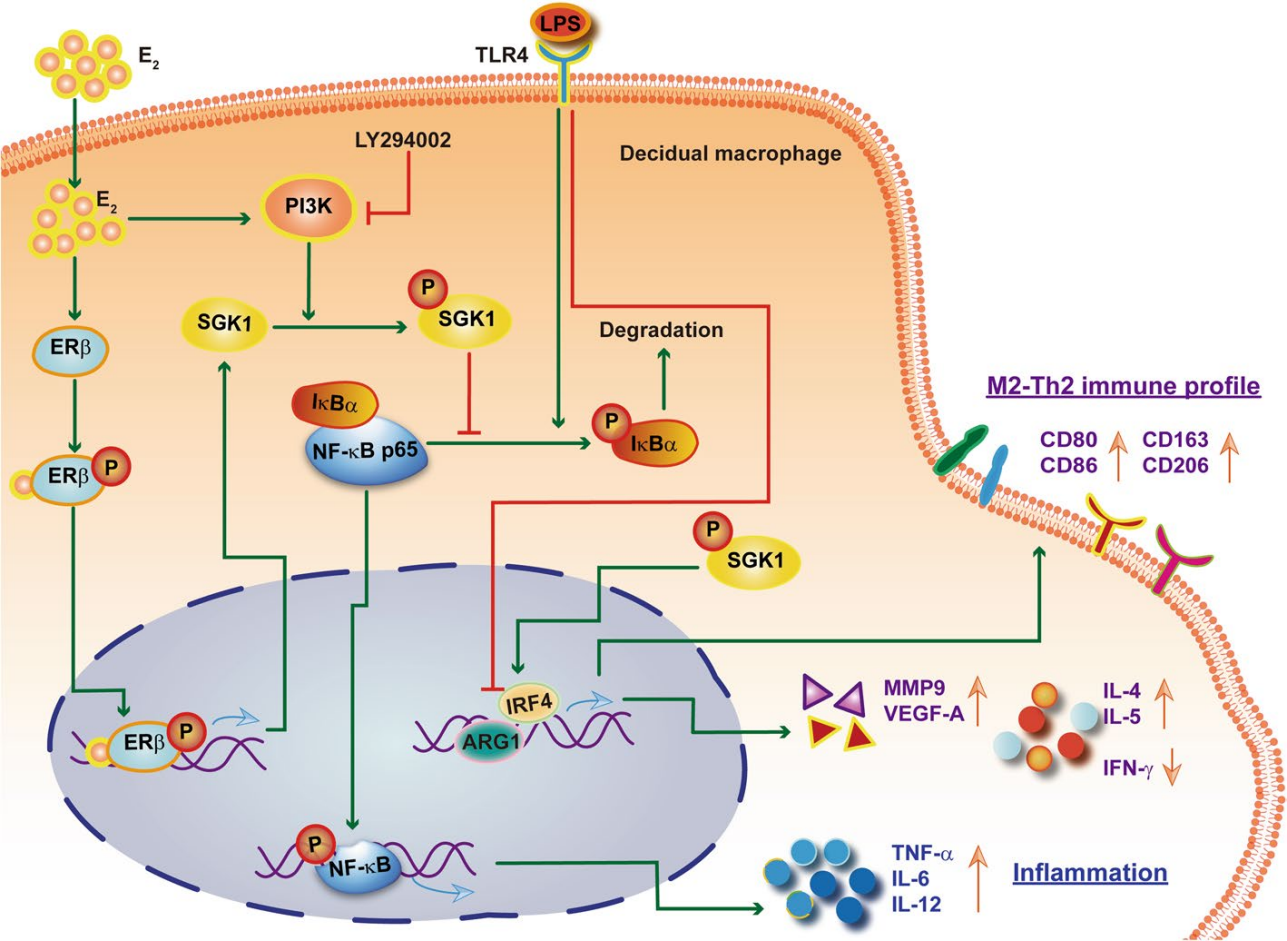
Schematic diagram of E_2_-activated SGK1 in priming anti-inflammatory M2 phenotype of macrophages at the maternal–fetal interface. Activation of SGK1 by E_2_ via the ERβ and PI3K signaling pathways elicits the M2 macrophage subtype, leading to elevated secretion of Th2 cytokines by increasing the expression levels of *ARG1* and *IRF4*. By contrast, SGK1 inhibition promotes pro-inflammatory cytokine productions by activating NF-κB in LPS-stimulated macrophages. Therefore, E_2_-sensitive activation of SGK1 contributes to anti-inflammatory M2 macrophage polarization and Th2 skewing at the maternal–fetal interface. E_2_, estradiol; SGK1, serum-glucocorticoid regulated kinase; PI3K, phosphoinositide 3-kinase; ER, estrogen receptor; NF-κB; nuclear factor kappa B; IκBα, inhibitor of nuclear factor kappa-B kinase subunit alpha; ARG1, arginase 1; IRF4, immune regulatory factor 4; LPS, lipopolysaccharide; IL, interleukin; IFN-γ, interferon gamma; MMP9, matrix metalloproteinase 9; VEGF-A, vascular endothelial growth factor A; TNF, tumor necrosis factor; IL, interleukin

We provide here evidence that the immunomodulatory role of E_2_-activated SGK1 in negatively regulating TLR-4-driven pro-inflammatory M1 polarization of macrophage and Th1 immune responses in the intrauterine microenvironment. Further investigation based on these results might provide a potential therapeutic target to manage RPL, especially in inflammation-related pregnancy loss (Fig. 7).

## Supplementary Information

**Additional file 1:** **Supplementary Figure S1.** E_2_ upregulates PGR, and P_4_ increases SGK1 activities in THP1 macrophages.

**Additional file 2:** **Supplementary Table S1.** Comparison of demographic characteristics of early pregnant women taking blood samples in this study. **Supplementary Table S2.** Comparison of the demographic characteristics of the studied women with RPL. **Supplementary Table S3.** Primers for quantitative real-time PCR. **Supplementary Table S4.** Information of primary antibodies used in western blotting analysis.

## Data Availability

The datasets used and analyzed during the current study are available from the corresponding author on reasonable request.

## Abbreviations

ARG1: Arginase 1
APC: Allophycocyanin
BCA: A bicinchoninic acid
CT: Cycle threshold
DAPI: 4’,6-Diamidino-2-phenylindole
DMEM: Dulbecco’s modified Eagle’s medium
DSCs: Decidual stromal cells
E_2_: 17ß-Estradiol
ER: Estrogen receptor
FBS: Fetal bovine serum
GAPDH: Glyceraldehyde-3-phosphate dehydrogenase
HESCs: Human endometrial stromal cells
HRP: Horseradish peroxidase
ICR: Institute of cancer research
IFN-$\gamma$: Interferon gamma
IκBα: Inhibitor of nuclear factor kappa-B kinase subunit alpha
IKK: Phosphorylating IκB kinase
IRF4: Immune regulatory factor 4
IL: Interleukin
LPS: Lipopolysaccharide
LSD: Least squares difference
MMP9: Matrix metalloproteinase 9
MPP: 1,3-Bis(4-hydroxyphenyl)-4-methyl-5-[4-(2-piperidinylethoxy)-phenol]-1H-pyrazole dihydrochloride
NF-κB: Nuclear factor kappa B
OVX: Ovariectomized
p-: Phospho-
P_4_: Progesterone
PBS: Phosphate-buffered saline
PE: Phycoerythrin
PerCP: Peridinin chlorophyll protein complex
PI3K: Phosphoinositide 3-kinase
PMA: Phorbol 12-myristate 13-acetate
PGR: Progesterone receptor
PVDF: Polyvinylidene fluoride
RIPA: Radioimmunoprecipitation assay
RPL: Recurrent pregnancy loss
SDS-PAGE: Sodium dodecyl sulfate–polyacrylamide gel electrophoresis
SEM: Standard error of mean
SGK1: Serum-glucocorticoid regulated kinase 1
siRNA: Small interfering RNA
SNK: Student–Newman–Keuls
TBP: TATA binding protein
Th: T helper
TLR4: Toll-like receptor
TNF: Tumor necrosis factor
VEGF-A: Vascular endothelial growth factor A
