# Supplementary material for: Estrogen-sensitive activation of SGK1 induces M2 macrophages with anti-inflammatory properties and a Th2 response at the maternal–fetal interface
Source: Reprod Biol Endocrinol. 2023 May 24;21:50. doi: 10.1186/s12958-023-01102-9 (PMC10207684; doi:10.1186/s12958-023-01102-9)
Supplement: Supplementary file 1 — Additional file 1: Supplementary Figure S1. E2 upregulates PGR, and P4 increases SGK1 activities in THP1 macrophages. [file 12958_2023_1102_MOESM1_ESM.docx]

**Supplementary Figure**


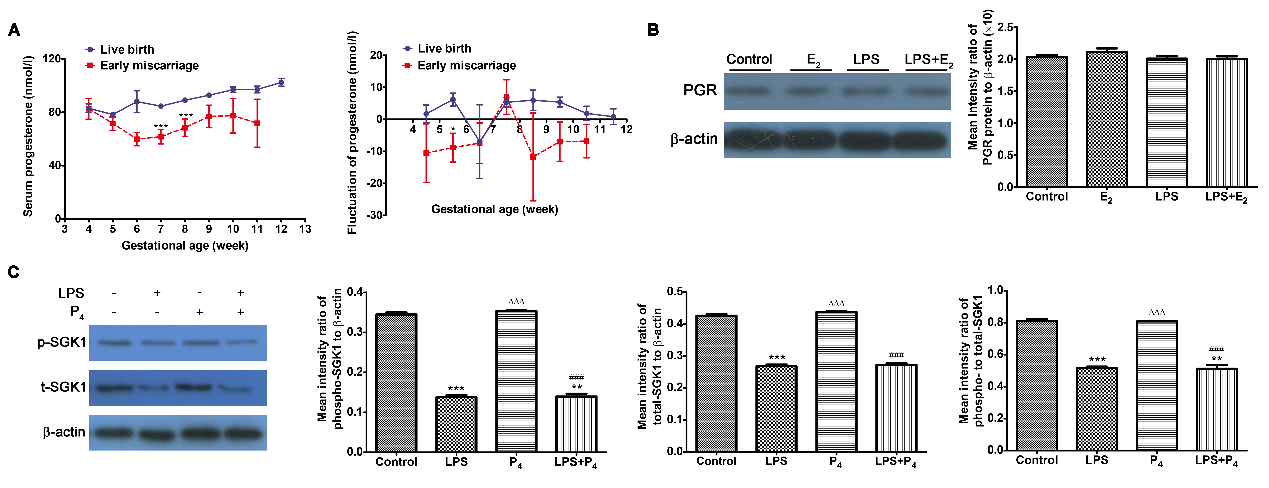


**Supplementary Figure S1. E_2_ upregulates** **PGR, and P_4_ increases SGK1 activities in THP‑1 macrophages**. (A) The concentrations (left) and the variation (right) of serum P_4_ (nmol/l) during the 4^th^–12^th^ week of gestation in a study population with live birth (n = 448) or miscarriage (n = 68). (B) Western blotting analysis of the PGR level relative to β-actin in THP-1 treated with LPS (10 ng/mL), E_2_ (10 nM), and LPS plus E_2_. (C) Western blotting analysis of p-SGK1, t-SGK1 in THP-1 treated with LPS (10 ng/mL), P_4_ (10 nM), and P_4_ plus LPS. Data are the arithmetic means ± SEM for three biological replicates. ***P* < 0.01, ****P* < 0.001, compared with control group or medium group; ∆∆∆*P* < 0.001, compared with LPS group; ###*P* < 0.001, compared with P_4_ group. E_2_, estradiol; PGR, progesterone receptor; P_4_, progesterone; SGK1, serum-glucocorticoid regulated kinase; OVX, ovariectomized; LPS, lipopolysaccharide; p-, phospho-; t-, total-; SEM, Standard Error of the Mean.
