# Supplementary material for: Estrogen-sensitive activation of SGK1 induces M2 macrophages with anti-inflammatory properties and a Th2 response at the maternal–fetal interface
Source: Reprod Biol Endocrinol. 2023 May 24;21:50. doi: 10.1186/s12958-023-01102-9 (PMC10207684; doi:10.1186/s12958-023-01102-9)
Supplement: Supplementary file 2 — Additional file 2: Supplementary Table S1. Comparison of demographic characteristics of early pregnant women taking blood samples in this study. Supplementary Table S2. Comparison of the demographic characteristics of the studied women with RPL. Supplementary Table S3. Primers for quantitative real-time PCR. Supplementary Table S4. Information of primary antibodies used in western blotting analysis. [file 12958_2023_1102_MOESM2_ESM.docx]

**Supplementary Tables**

**Supplementary Table S1.** Comparison of demographic characteristics of early pregnant women taking blood samples in this study.

| Variables | Live birth  (n=448) | Early miscarriage  (n=68) | *P* value |
| --- | --- | --- | --- |
| Age, years | 30.00 ± 0.17 | 30.40 ± 0.52 | .410 |
| Total number of abortions | 1.48 ± 0.06 | 1.81 ± 0.23 | .078 |
| Previous miscarriages | 0.73 ± 0.04 | 0.96 ± 0.16 | .050 |

Notes: Independent samples t-test was used. Data are expressed as mean ± SEM. *P* < .05 considered statistically significant.

**Supplementary Table S2.** Comparison of the demographic characteristics of the studied women with RPL.

| Variables | Normal pregnancy  (n=66) | RPL  (n=93) | *P* value |
| --- | --- | --- | --- |
| Age, years | 27.89 ± 0.74 | 29.43 ± 0.48 | .071 |
| BMI, kg/m^2^ | 20.79 ± 0.30 | 21.11 ± 0.31 | .474 |
| Gestational age, weeks | 7.64 ± 0.14 | 7.92 ± 0.14 | .159 |
| Parity (previous births) | 0.50 ± 0.07 | 0.19 ± 0.04 | < .001 *** |
| Previous induced abortion | 0.00 ± 0.00 | 0.61 ± 0.08 | < .001 *** |
| Serum E_2_ (pg/ml) | 1026.44 ± 50.64 | 474.09 ± 34.68 | < .001 *** |
| *SGK1* expression (2^–ΔCT^) | 0.30 ± 0.03 | 0.19 ± 0.02 | < .001 *** |

Notes: RPL, recurrent pregnancy loss; BMI, body mass index; SGK1, serum-glucocorticoid regulated kinase 1. Independent samples t-test was used. Data are presented as mean ± SEM. ***, *P* < .001.

**Supplementary Table S3.** Primers for quantitative real-time PCR.

| Genes | GenBank accession number | Primer sequence (5’-3’) | Product size (bp) |
| --- | --- | --- | --- |
| Human SGK1 | NM_001143676.1 | TCATGCCAACATCCTGACCAA (Forward) | 102 |
|  |  | TGAATAAAGTCGTTCAGACCCATCC (Reverse) |  |
| Human IRF4 | U52682 | CCAAGATTCCAGGTGACTC (Forward) | 176 |
|  |  | GGATTGCTGATGTGTTCTG (Reverse) |  |
| Human ARG1 | NM_001244438 | CCACAGTTTGGCAATTGGAA (Forward) | 75 |
|  |  | GCATCCACCCAGATGACTCC (Reverse) |  |
| Human MMP9 | NM_004994.2 | GAGGCGCTCATGTACCCTATGT (Forward) | 101 |
|  |  | GGTTCAGGGCGAGGACCATA (Reverse) |  |
| Human VEGF-A | NM_001204384 | AATGTGAATGCAGACCAAAG (Forward) | 106 |
|  |  | GACTTATACCGGGATTTCTTG (Reverse) |  |
| Human GAPDH | NM_002046.3 | GCACCGTCAAGGCTGAGAAC (Forward) | 138 |
|  |  | TGGTGAAGACGCCAGTGGA (Reverse) |  |

Notes: SGK1, serum-glucocorticoid regulated kinase 1; IRF4, immune regulatory factor 4; ARG1, arginase 1; MMP9, matrix metalloproteinase 9; VEGF-A, vascular endothelial growth factor A; GAPDH, glyceraldehyde-3-phosphate dehydrogenase.

**Supplementary Table S4.** Information of primary antibodies used in western blotting analysis.

| **Primary antibodies** | **Catalogue Number** | **Supplier** | **Dilution** | **Application** |
| --- | --- | --- | --- | --- |
| p-SGK1 | sc-16744 | Santa Cruz Biotechnology | 1:800 | WB in THP1 |
| SGK1 | sc-28338 | Santa Cruz Biotechnology | 1:800 | WB in THP1 |
| p-IκBα | sc-8404 | Santa Cruz Biotechnology | 1:500 | WB in THP1 |
| IκBα | sc-847 | Santa Cruz Biotechnology | 1:500 | WB in THP1 |
| p-NF-κB p65 | #3033 | Cell Signaling Technologies | 1:1000 | WB in THP1 |
| NF-κB p65 | #3034 | Cell Signaling Technologies | 1:1000 | WB in THP1 |
| ESR2 | ab133467 | Abcam | 1:1000 | WB in THP1 |
| β-actin | sc-47778 | Santa Cruz Biotechnology | 1:1500 | WB in THP1 |
| TBP | ab197874 | Abcam | 1:5000 | WB in THP1 |
| p-SGK1 | ab55281 | Abcam | 1: 2000 | WB in mice |
| SGK1 | sc-28338 | Santa Cruz Biotechnology | 1: 500 | WB in mice |
| p-IκBα | CST 2859 | Cell Signaling Technologies | 1: 1000 | WB in mice |
| IκBα | CST 4814 | Cell Signaling Technologies | 1: 1000 | WB in mice |
| p-NF-κB p65 | CST 3033 | Cell Signaling Technologies | 1: 1000 | WB in mice |
| NF-κB p65 | CST 8242 | Cell Signaling Technologies | 1: 1000 | WB in mice |
| β-actin | ab8226 | Abcam | 1: 1000 | WB in mice |
| TBP | ab818 | Abcam | 1: 2000 | WB in mice |

Notes: p-, phospho-; SGK1, serum-glucocorticoid regulated kinase 1; IκBα, inhibitor of nuclear factor kappa-B kinase subunit alpha; NF-κB; nuclear factor kappa B; ESR2, gene encodes estrogen receptor beta, TBP, TATA binding protein.
